# Comparison of currently common neoadjuvant therapy strategies for rectal cancer: a three-arm retrospective study

**DOI:** 10.3389/fcimb.2025.1545195

**Published:** 2025-12-09

**Authors:** Xiao Zhang, Yang An, Yuxin Liu, Ganbin Li, Xiaoyuan Qiu, Yihan Lu, Guole Lin

**Affiliations:** Department of General Surgery, Peking Union Medical College Hospital, Chinese Academy of Medical Sciences & Peking Union Medical College, Beijing, China

**Keywords:** neoadjuvant therapy, rectal cancer, rectal MRI, restage, gut microbiome

## Abstract

**Background:**

The evolving neoadjuvant therapy regime is revolutionizing the management of local advanced rectal cancer (LARC). Total neoadjuvant therapy (TNT) and neoadjuvant immunotherapy are currently the most prominent strategies. However, existing studies predominantly evaluate these approaches in isolation, leaving their comparative efficacy unresolved.

**Methods:**

A three-arm retrospective study was conducted including a total of 160 consecutive patients pathologically diagnosed as adenocarcinoma with pMMR or non-MSI-H and receiving neoadjuvant therapy followed by radical resection in Peking Union Medical College Hospital (PUMCH). Based on the neoadjuvant therapy regimen, patients were divided into three groups: the nCRT group (n=81), the TNT group (n=42), and the PD-1 group (n=37). The clinical data including baseline characteristics, treatment information, and MRI accuracy on rectal cancer restaging were analyzed. Considering the possible impact of gut microbiome on antitumor immunity, we also analyzed differences in gut microbiome between baseline stool samples from pCR and non-pCR patients in the PD-1 group.

**Results:**

No significant differences were found in baseline characteristics among the three groups. The rates of pathologic complete response (pCR, corresponding to pTRG 0) were 25.9% (21/81) in the nCRT group, 40.5% (17/42) in the TNT group, and 45.9% (17/37) in the PD-1 group (p=0.048). The accuracy of MRI for restaging rectal cancer T stage was not ideal, particularly in the TNT and PD-1 groups. Additionally, the α and β diversity of gut microbiome between baseline stool samples from pCR and non-pCR patients in the PD-1 group were similar.

**Conclusions:**

Both TNT and PD-1 combination therapy demonstrated higher tumor regression and pCR rates compared with nCRT, suggesting enhanced local tumor control. However, improvements in rectal MRI accuracy and gut microbiome research are needed to enhance precision in diagnostics and therapy.

## Introduction

The management of locally advanced cancer (LARC) remains a significant clinical challenge for colorectal surgeons. Over the past few decades, multimodal therapy, which includes neoadjuvant therapy, surgery and postoperative chemotherapy, has become the standard treatment approach for patients with LARC (Berardi et al., 2014; Koukourakis et al., 2023). The application of neoadjuvant therapy prior to the surgery aims to downstage the tumor, increase resectability, reduce local recurrence rates, and optimize oncology outcomes (Body et al., 2021; Ominelli et al., 2021). Additionally, some patients may achieve a pathological or clinical complete response (CR) after neoadjuvant therapy, resulting in better oncological outcomes and the potential for non-operative management (Cercek et al., 2022).

However, the considerable distance recurrence rate and low CR rate of conventional neoadjuvant therapy highlight the need for new strategies in treating LARC. Recently, emerging strategies, such as total neoadjuvant therapy (TNT) and neoadjuvant immunotherapy are transforming treatment paradigms. TNT delivers systemic chemotherapy before the surgery, aiming to eliminate micrometastatic disease early, enhance antitumor activity, and improve patient compliance with systemic therapy. Many clinical trials have demonstrated higher pCR rate with TNT compared to traditional neoadjuvant chemoradiotherapy (nCRT). For instance, the RAPIDO trial showed that TNT significantly reduced disease-related treatment failure (23.7% vs. 30.4%) compared with standard nCRT, while doubling the pCR rate (28% vs. 14%). Similarly, the PRODIGE-23 trial demonstrated that induction FOLFIRINOX followed by chemoradiotherapy achieved a higher pCR rate (28% vs. 12%) and improved 3-year disease-free survival (76% vs. 69%) relative to the conventional approach (Bahadoer et al., 2021; Conroy et al., 2021; Jin et al., 2022).

Neoadjuvant immunotherapy, particularly with immune checkpoint inhibitors, has shown promise, especially in tumors with mismatch repair deficiency (dMMR) or high microsatellite instability (MSI-H), which are associated with a high mutational burden and increased immunogenicity (Cercek et al., 2022; Zhang, 2022). The combination of immunotherapy with other modalities, such as radiotherapy and chemotherapy can create a synergistic effect and amplify the therapeutic efficacy of each other. There is also encouraging evidence that applying immunotherapy to mismatch repair-proficient (pMMR) tumors may improve pCR rates and other oncological outcomes (Mi et al., 2022; Yang et al., 2024a). The phase II SPRING-01 trial found that sintilimab combined with short-course radiotherapy and CapeOX chemotherapy significantly improved the pCR rate to 59.2% compared with 32.7% in the control group, without introducing unacceptable toxicity. Moreover, the CHOICE-01 trial explored the efficacy of PD-1 inhibitor–based neoadjuvant chemoradiotherapy in patients with ultra-low rectal cancer, achieving a clinical complete response (cCR) rate of 45.5% and rectal preservation in 63.4% of patients (Tian et al., 2025; Zhou et al., 2025).

Despite growing interest in these novel approaches, existing evidence predominantly evaluates each strategy in isolation rather than within a unified comparative framework. Specifically, whether PD-1 inhibitors combined with nCRT offer additional benefits compared with the sequential chemotherapy–radiation paradigm of TNT remains uncertain. Clarifying this issue is essential for guiding optimal treatment selection and advancing precision therapy tailored to tumor biology and disease stage. Therefore, we conducted this retrospective study to compare the efficacy of three common neoadjuvant therapy regimes (traditional nCRT vs. nCRT combined with PD1 inhibitor vs. TNT) for patients with LARC in clinical practice. By exploring their relative advantages in specific clinical scenarios, this research aims to inform evidence-based therapeutic selection and guide the design of future randomized trials in LARC management. Additionally, considering that the gut microbiome may influence antitumor immunity through metabolites and affect responses to radiotherapy and immunotherapy, we also investigated the relationship between immunotherapy efficacy and the gut microbiome.

## Methods

This single-center retrospective study enrolled patients who were initially diagnosed with rectal adenocarcinoma and started neoadjuvant therapy in Peking Union Medical Hospital from Aug 2022 to Aug 2023. The study has been approved by the Ethics Committee of Peking Union Medical College Hospital, with approval number I-22YJ405.

Inclusion criteria were as follows: 1) Patients aged 18 to 75 years with ECOG score< 2; 2) Pathologic diagnosis of adenocarcinoma with proficient mismatch repair (pMMR) or non-MSI-H status; 3) Diagnosis of locally advanced rectal cancer based on rectal MRI (cT3–4 or N1-2), with the tumor located within 10 cm of the anal verge; 4) Patients who completed a full course of neoadjuvant therapy followed by radical resection; 5) Patients with clinically staged M0 disease (absence of distant metastasis) confirmed by contrast-enhanced CT of the chest, abdomen, and pelvis; 6) No history of other cancers or prior anti-tumor treatments. Exclusion criteria is as follows: 1) Patients diagnosed with multiple cancers or concurrent malignant tumors aside from rectal cancer; 2) Patients who refused surgery or opted local resection after neoadjuvant therapy; 3) Patients who discontinued neoadjuvant therapy midway due to adverse effects or other reasons; 4) Patients who did not undergo rectal MRI evaluation before or after neoadjuvant therapy or did not have a standardized MRI report; 5) Patients who received a neoadjuvant regimen other than conventional nCRT, TNT, or neoadjuvant immunotherapy; 6) Patients with incomplete and untraceable clinical data.

The selection of neoadjuvant therapy regimens was determined through shared decision-making, incorporating physician expertise, multidisciplinary recommendations and patient-centered preference. As an observational retrospective study, treatment allocation strictly reflected real-world clinical practice without researcher influence. Patients will be categorized into three groups based on their neoadjuvant therapy regimen: the nCRT group, the TNT group, and the PD-1 group. The specific treatment protocols for each group were as follows:

1) nCRT group: Patients received conventional nCRT regime, which involves a long course of radiotherapy (50 Gy in 25 fractions, 5 fractions per week) synchronized with orally capecitabine (825 mg/m^2^, orally, twice daily). Capecitabine was administered throughout the radiotherapy course, with dose reductions (20-50%) permitted for grade ≥3 hematologic or gastrointestinal toxicity. Surgery is scheduled to occur 8 to 12 weeks after the completion of radiotherapy.

2) TNT group: Patients first received concurrent chemoradiotherapy which is same as the nCRT group but followed with 4–6 courses of consolidation chemotherapy (XELOX regime, 130 mg/m2 of oxaliplatin intravenously on day 1 plus oral capecitabine 1,000 mg/m2, twice daily from day 1 to day 14, 21 days per course). Consolidation chemotherapy commenced 2 weeks post-radiotherapy completion. And Radical surgery is performed around 2 weeks post-chemotherapy for recovery of bone marrow and organ function.

3) PD-1 group: Patients received concurrent chemoradiotherapy identical to the nCRT group. Additionally, three 21-day cycles tislelizumab (200 mg, iv.gtt, day1), which is a common PD1 inhibitor, were administered in the next nine consecutive weeks. The first dose was given on Day 8–10 of radiotherapy. The operation also took place within 8–12 weeks after the completion of radiotherapy.

All surgeries were performed by experienced colorectal surgeons adhering to total mesorectal excision (TME) principles. Pathological assessments were conducted by gastrointestinal-specialized pathologists.

The primary endpoint of this retrospective analysis was to evaluate the pathological response among three groups. American Joint Committee on Cancer (AJCC, eighth edition) tumor regression grade (TRG) system was applied to assess the tumor response to neoadjuvant therapy. Secondary endpoints included the accuracy of MRI-based restaging, evaluated by comparing restaging MRI after neoadjuvant therapy with final pathological staging, as well as the neoadjuvant rectal (NAR) score, which is a predictor of survival after neoadjuvant therapy for rectal cancer. NAR score is calculated using the following formula and categorized as low (<8), intermediate (8-16), or high (>16). In the formula, ypN and ypT represent the pathologic N-stage and T-stage of the tumor, respectively, while cT refers to the baseline T-stage of the tumor as assessed by rectal MRI prior to neoadjuvant therapy (Mukai et al., 2020; Glynne-Jones and Glynne-Jones, 2021; Imam et al., 2021). All the clinical data of the three groups were collected through the Electronic Medical Record (EMR) System in our hospital. The study variables included basic clinical features and treatment-related information. Basic clinical features comprised sex, age, body mass index (BMI), history of chronic diseases such as hypertension (HT), diabetes mellitus (DM) and chronic heart diseases (CHD), ASA_PS scores, history of smoking and drinking, baseline carcinoembryonic antigen (CEA) value and tumor assessment data based on rectal MRI. Treatment related information included time intervals between radiotherapy and TME, operation type, TME quality and pathological diagnostic data.


$$\text{NAR}=\frac{{\left[5ypN-3\left(cT-ypT\right)+12\right]}^{2}}{9.61}$$

In addition, the fecal samples collected before the neoadjuvant therapy of 22 patients in PD-1 group, which were stored at -80°C in the laboratory, were analyzed in this study. Total genome DNA from samples was extracted using CTAB/SDS method. The 16S rRNA genes (V4/V3/V3-V4/V4-V5 regions) were amplified by PCR using region-specific primers (e.g., 515F-806R for V4) with barcodes. Amplification employed Phusion^®^ High-Fidelity PCR Master Mix, followed by product purification using the Qiagen Gel Extraction Kit. Libraries were prepared using the TruSeq^®^ DNA PCR-Free Sample Preparation Kit, assessed with Qubit^®^ 2.0 Fluorometer and Agilent Bioanalyzer 2100 system, and sequenced on the Illumina NovaSeq platform with 250 bp paired-end reads.

As this was a retrospective real-world study, the sample size was determined by the availability of eligible patients rather than by *a priori* calculation. Statistical analyses were performed using SPSS software (version 25.0), GraphPad software (version 8.3.0) and R (version 4.2). Normally distributed continuous variables were expressed as mean ± standard deviation (SD) and analyzed using one-way ANOVA, while non-normally distributed variables were presented as median (Q1, Q3) and compared using the Kruskal-Wallis test. Categorical variables were summarized as frequencies and percentages and evaluated with the Chi-square test or Fisher’s exact test when appropriate. All analyses were two-tailed with a 95% confidence interval, and statistical significance was defined as P< 0.05. To account for multiple comparisons, the Holm–Bonferroni correction was applied in *post hoc* analyses.

## Results

A total of 160 patients were finally enrolled and analyzed in this retrospective study of which 105 were males. The mean age was 59.36 and the median BMI was 24.28. The patients were divided into three groups: 81 in the nCRT group, 42 in the TNT group, and 37 in the PD-1 group. There were no significant differences, among the patients in the three groups in the demographical parameters, such as age, sex, and BMI. In terms of baseline tumor assessment, the distance from the tumor to the anal verge measured 6.12 ± 2.27 cm for the nCRT group, 6.46 ± 2.17 cm for the TNT group, and 6.35 ± 2.25 cm for the PD-1 group. The sagittal length of the tumors was 4.11 ± 1.56 cm for the nCRT group, 4.40 ± 1.36 cm for the TNT group, and 4.13 ± 1.35 cm for the PD-1 group; no significant differences were noted. Additional details regarding the basic characteristics of the three groups are shown in **Table 1**.

**Table 1:** Baseline characteristics.

Variables	nCRT (n = 81)	TNT group (n = 42)	PD1 group (n = 37)	*P*
male, n (%)	48 (59.26)	32 (76.19)	25 (67.57)	0.166
age, y, Mean ± SD	59.68 ± 11.93	59.74 ± 8.57	58.22 ± 11.72	0.776
BMI, kg/m2, M (Q_1_, Q_3_)	24.49 (22.50,26.57)	23.44 (21.38,24.88)	24.35 (21.80,26.64)	0.173
HT, n (%)	33 (40.74)	12 (28.57)	11 (29.73)	0.303
DM, n (%)	15 (18.52)	5 (11.90)	8 (21.62)	0.496
CHD, n (%)	5 (6.17)	3 (7.14)	0 (0.00)	0.348
abdominal surgical history, n (%)	12 (14.81)	8 (19.05)	11 (29.73)	0.164
ASA_PS, n (%)				0.435
1	8 (9.88)	1 (2.38)	2 (5.41)	
2	65 (80.25)	35 (83.33)	33 (89.19)	
3	8 (9.88)	6 (14.29)	2 (5.41)	
smoke history, n(%)	25 (30.86)	21 (50.00)	10 (27.03)	0.055
alcoholic history, n(%)	22 (27.16)	17 (40.50)	8 (21.62)	0.153
baseline CEA, ng/mL, M (Q_1_, Q_3_)	3.75 (2.10,8.62)	4.55 (2.35,13.30)	3.00 (2.00,10.00)	0.393
tumor distance to the anal verge, cm, Mean ± SD	6.12 ± 2.27	6.46 ± 2.17	6.35 ± 2.25	0.691
Sagittal diameter length of the tumor measured on MRI, cm, Mean ± SD	4.11 ± 1.56	4.40 ± 1.36	4.13 ± 1.35	0.553
mrT stage, n(%)				0.561
T1-T2	11 (13.58)	3 (7.14)	4 (10.81)	
T3-T4	70 (86.42)	39 (92.86)	33 (89.19)	
mrN stage, n(%)				0.812
N0	6 (7.41)	2 (4.76)	3 (8.11)	
N+	75 (92.59)	40 (95.24)	34 (91.89)	
MRF, n(%)				0.38
negative	46 (56.79)	23 (54.76)	16 (43.24)	
positive	35 (43.21)	19 (45.24)	21 (56.76)	
EMVI, n(%)				0.925
negative	40 (49.38)	21 (50.00)	17 (45.95)	
positive	41 (50.62)	21 (50.00)	20 (54.05)	

SD: Standard deviation, M: Median, Q1: First Quartile, Q3: Third Quartile,

HT: hypertension, DM: diabetes mellitus, CHD: coronary heart disease, ASA_PS: American Society of Anesthesiologists Physical Status

*: statistical significance

All patients underwent radical resection after neoadjuvant therapy, with only two patients (2.5%) in the nCRT group, two patients (4.8%) in the TNT group, and three patients (8.9%) in the PD-1 group failing to preserve the anal. The proportion of patients with specimens assessed as complete was 86.42%, 88.10%, and 91.89% across the three groups, respectively, with no statistical differences. Regarding the response to neoadjuvant therapy, the distribution of pTRG evaluations across the three groups was statistically different. A higher percentage of patients in the PD-1 group (45.95%) and the TNT group (40.48%) were assessed as TRG 0, compared to the nCRT group (25.93%). However, in the *post-hoc* analysis, only the difference between the TNT group and the nCRT group was confirmed as statistically significant. There was no statistically significant difference in the NAR score among the three groups (**Table 2**).

**Table 2:** Treatment outcomes.

Variables	Cohorts			Statistic	*P-value*	Post-hoc analysis (*p-value)*		
	nCRT group (n = 81)	TNT group (n = 42)	PD1 group (n = 37)			nCRT vs TNT	nCRT vs PD-1	TNT vs PD-1
operation type, n (%)				χ²=1.951	0.377	N/C	N/C	N/C
anal-preserving surgery	79 (97.53)	40 (95.24)	34 (91.89)					
not anal-preserving surgery	2 (2.47)	2 (4.76)	3 (8.11)					
TME quality					0.763	N/C	N/C	N/C
complete	70 (86.42)	37 (88.10)	34 (91.89)					
near complete	10 (12.34)	4 (9.52)	3 (9.11)					
incomplete	1 (1.24)	1 (2.38)	0					
pT stage				χ²=9.11	0.168	N/C	N/C	N/C
0	21 (25.93)	17 (40.48)	17 (45.95)					
1	7 (8.64)	2 (4.76)	3 (8.11)					
2	24 (29.63)	6 (14.29)	9 (24.32)					
3	29 (35.80)	17 (40.48)	8 (21.62)					
pN stage					0.527	N/C	N/C	N/C
N0	62 (76.54)	34 (80.95)	32 (86.49)					
N1	16 (19.75)	7 (16.67)	5 (13.51)					
N2	3 (3.71)	1 (2.38)	0					
pTRG				-	0.016*	0.019(α adj = 0.025)	0.017 (α adj = 0.017)	0.809(α adj = 0.0.05)
0	21 (25.93)	17 (40.48)	17 (45.95)					
1	25 (30.86)	12 (28.57)	12 (32.43)					
2	34 (41.98)	9 (21.43)	6 (16.22)					
3	1 (1.23)	4 (9.52)	2 (5.40)					
Nerve invasion, n (%)	6 (7.41)	5 (11.90)	2 (5.41)		0.542	N/C	N/C	N/C
Lymphovascular invasion, n (%)	2 (2.47)	2 (4.76)	1 (2.70)		0.775	N/C	N/C	N/C
NARscore								
<8	28 (34.57)	19 (45.24)	17 (45.95)		0.252	N/C	N/C	N/C
8-16	38 (46.91)	17 (40.48)	17 (45.95)					
>16	15 (18.52)	6 (14.28)	3 (8.10)					

*statistical significance.

**Figure 1:**
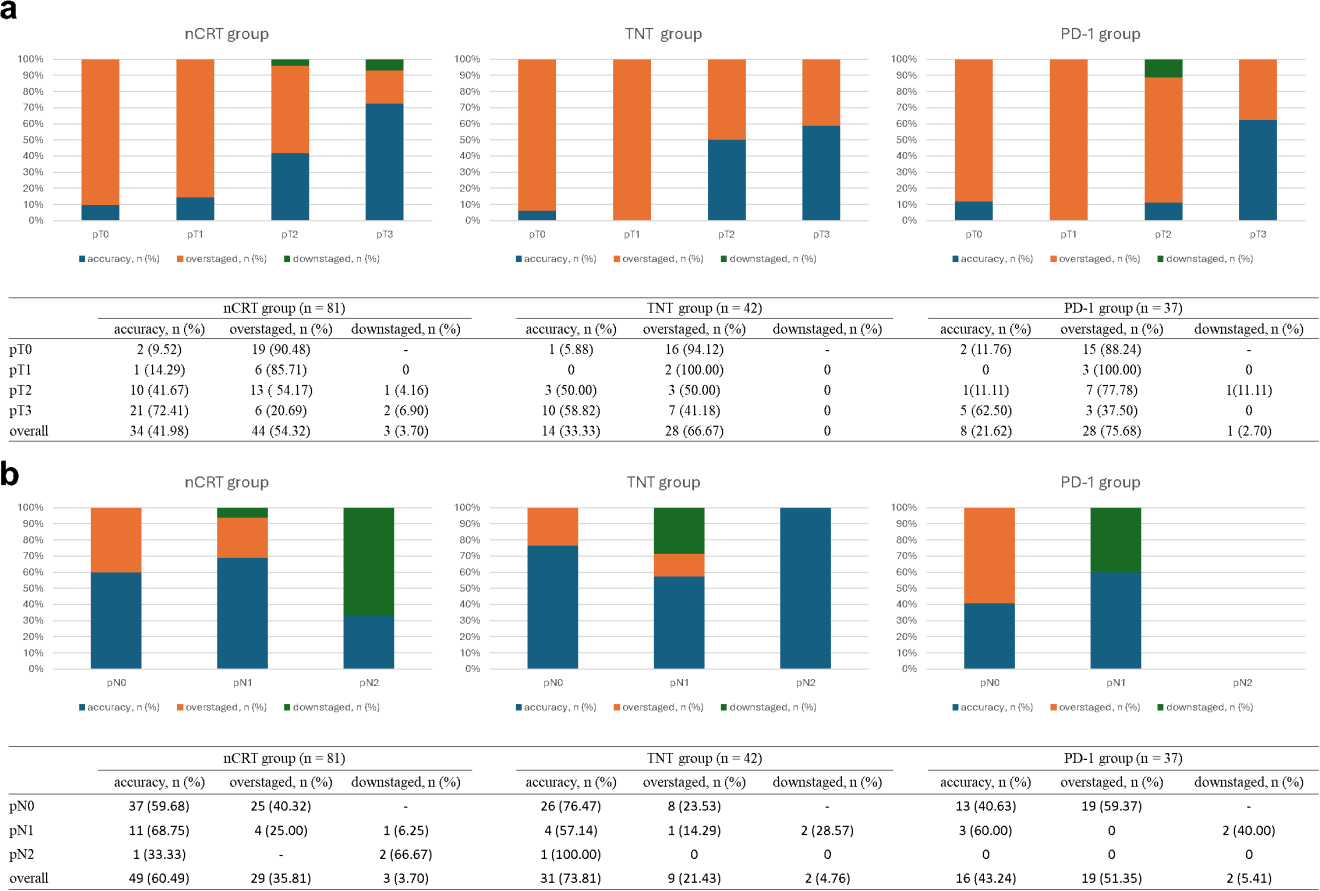
Accuracy of rectal MRI on the restaging of tumor after neoadjuvant therapy. **(a)** shows the accuracy of MRI for reassessing T-stage in three groups; **(b)** shows the accuracy of MRI for reassessing N-stage in three groups.

As shown in **Figure 1**, we evaluated differences in the accuracy of MRI restaging after receiving different neoadjuvant treatment regimens. The accuracy of MRI for reassessing T staging was under 50% in all three groups, and it appeared to be lower in the TNT and PD-1 groups compared to the nCRT group. Notably, patients staged as pT3 had higher accuracy rates regardless of the neoadjuvant therapy received. For N stage, the accuracy of MRI was 60.49% in the nCRT group, 73.81% in the TNT group, and 43.24% in the PD-1 group, representing relatively better results compared to the reassessment for T staging.

**Figure 2:**
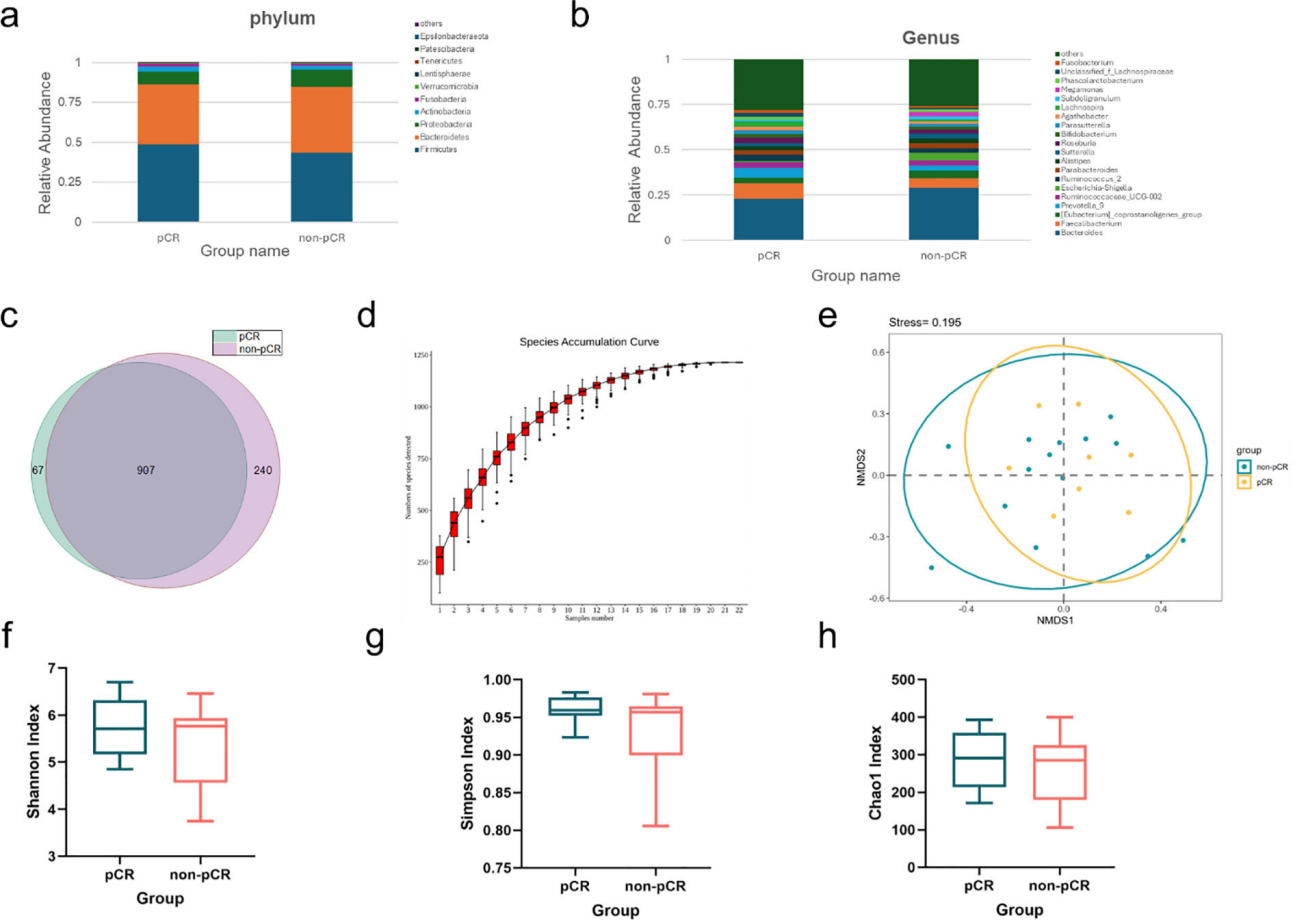
Microbial composition and diversity of patients who achieved pCR or non-pCR in the PD-1 group: **(a, b)** Bar plot presents the relative abundance on the phylum, and genus levels in pCR and non-pCR patients; **(c)** Amplicon Sequence Variants (ASVs) between pCR and non-pCR patients; **(d)** Cumulative plots of relative species abundance reflect the relationship between sample size and the number of species. The flattening of the curve indicates adequate sampling; **(e)** Non-metric multidimensional scaling (NMDS) plot visualizes the overall microbiome dissimilarity measured by Bray–Curtis dissimilarities; **(f–h)** Shannon, Simpson, and Chao1 index of pCR and non-pCR patients (p > 0.05).

Of the 22 patients in the PD1 group who underwent fecal testing, 8 patients achieved pCR. Sequencing and quality control were performed to obtain high-quality reads for subsequent analysis on the Illumina NovaSeq platform. The results were shown in **Figure 2**. An average of 79,997 raw reads was generated per sample, and an average of 71,930 clean reads was retained after quality control, with a quality control efficiency of 89.9%. Amplicon Sequence Variants (ASVs) were identified using the DADA2 pipeline. A total of 1214 ASVs were detected, including 974 ASVs in the pCR group and 1,147 ASVs in the non-pCR group. For further analysis, the representative ASV sequences was annotated using the *classify-sklearn* method (Naive Bayes). At the phylum level, the most abundant compositions of pCR group and non-pCR group were both Firmicutes, Bacteroidetes, Proteobacteria, Actinobacteria, and Fusobacteriota. Microbial community richness and diversity (α-diversity) were measured, and enrichment curves were shown in **Figure 2d**. Chao1 index, Shannon index and Simpson index of pCR and non-pCR patients were not significantly different. In terms of the overall microbiota (β-diversity) between the two groups, PERMANOVA analysis based on the Bray–Curtis dissimilarity (**Figure 2e**), weighted, and unweighted UniFrac (**Supplementary Figure S1**) were conducted and found no significant differences.

## Discussion

Neoadjuvant therapy has been recognized as crucial component of multimodal therapy for LARC (Oronsky et al., 2020; Koukourakis et al., 2023). Investigating effective neoadjuvant therapy regimens to enhance tumor response prior to surgery and improve oncological outcomes is a prominent focus in clinical practice (Boublikova et al., 2023; Li et al., 2024; Yang et al., 2024a). In this single-center, three-arm retrospective study, we compared three most common neoadjuvant therapy regimens for LARC including traditional neoadjuvant therapy (nCRT group), TNT (TNT group) and neoadjuvant chemoradiotherapy combined with PD-1 inhibitors (PD-1 group). The results indicated that both TNT and the combination with PD-1 inhibitors could lead to improved tumor response. Although long-term outcomes were not yet available, no statistically significant differences in NAR scores were observed among the three groups. These results provide preliminary evidence that should be further validated through studies with extended follow-up to clarify their long-term prognostic value.

TNT is an emerging neoadjuvant therapy regime for LARC and has been accepted as a standard treatment in recent years. Several randomized controlled trials, such as RAPIDO (Bahadoer et al., 2021) and PRODIGE-23 (Conroy et al., 2021; Conroy et al., 2024), have shown that the main advantages of TNT include improved pathological complete response rates, reduced distant metastases, and potentially increased overall survival. Our study also confirmed that compared to nCRT, TNT enhances tumor response. It’s important to note that the specific regimen of TNT is not unique. For instance, in terms of chemotherapy, which is brought forward in TNT, there are mainly two main timelines known as induction (delivering systemic chemotherapy before radiotherapy) and consolidation (delivering systemic chemotherapy after CRT) (Ominelli et al., 2021). Preoperative radiotherapy could also be divided into two options at least: long-course and short-course radiotherapy (Ngan et al., 2012). In our study, all patients in the TNT group received the same protocol (See “Methods” section), which is the preferred TNT regimen at our center. The differences between different TNT regimes were not compared in our trial.

The role of immunotherapy, particularly immune checkpoint inhibitors, in the neoadjuvant treatment of LARC is increasingly significant (Yang et al., 2022). While the effectiveness of PD-1 inhibitors in patients with pMMR and non-MSI-H is not satisfactory, multiple studies have validated that combining chemoradiotherapy with PD-1 inhibitors can yield better clinical efficacy and acceptable safety (Xia et al., 2024; Yang et al., 2024a). Radiotherapy may enhance the therapeutic effect of PD-1 inhibitors by promoting the immune response (Yang, 2022). However, the optimal regimen for this combination and the appropriate patient selection remains unclear and needs a lot of work. Our findings suggest that tumor regression rates were similar in the TNT and PD-1 groups. To our knowledge, there have been no clear reports comparing the efficacy of these two strategies. Therefore, accurately identifying patient populations that may benefit from different treatment options, using various strategies such as liquid biopsies, is becoming increasingly important (Aschele and Glynne-Jones, 2023). In addition, cost-effectiveness is a critical factor in the evaluation of new treatment strategies. Re-I Chin’s team suggested that TNT incorporating short-course radiotherapy followed by TME may represent a novel, cost-saving treatment paradigm for the management of LARC compared to traditional nCRT (Chin et al., 2022). However, there is currently a lack of research specifically assessing the cost-effectiveness of TNT, immunotherapy-based approaches, and traditional nCRT. We believe that further high-quality studies are needed to provide comprehensive economic evaluations and guide evidence-based decision-making in clinical practice.

With improvements in tumor response to neoadjuvant therapy, some patients achieving a complete response (CR) or near-complete response (nCR) can be spared from surgery or receive only local resection, which contributes to the preservation of anal function and improvement of quality of life. Consequently, accurate assessment of CR and nCR based on clinical evaluations before surgery has gained increased attention. Rectal MRI is a standard method for restaging rectal cancer after neoadjuvant therapy and is widely utilized in clinical practice. However, the varying therapeutic intensities and improvements in tumor response may affect the assessment of MRI (Gefen et al., 2023). We found that T stage is more likely to be overstated in the TNT group and the PD-1 group, particularly among patients exhibiting stronger tumor responses. This may be related not only to the technical limitations of MRI but also to the conservation and lack of experience in restaging. Current standards rely on various methods, including colonoscopy, MRI, digital rectal examination, and other evaluations. Yet, the accuracy of identifying cCR and nCR still requires improvement.

The gut microbiome, comprising trillions of microorganisms residing within the gastrointestinal tract, plays a critical role in maintaining human health and disease. Some studies have suggested that the gut microbiome may significantly influence the efficacy of anti-PD-1 immunotherapy in melanoma, hepatocellular carcinoma and many other cancer (Gopalakrishnan et al., 2018; Routy et al., 2018; Zheng et al., 2019). Specific microbial signatures and diversity within the gut microbiome have been correlated with enhanced immune activation and improved therapeutic responses, underscoring the microbiome’s potential as a biomarker for patient stratification and a modulator of treatment outcomes. Yuxi Yi et al. found that the gut microbiome may serve as a novel potential biomarker for predicting response to nCRT, and significant alterations in the gut microbiome were observed during the course of treatment (Yi et al., 2021). Similarly, Zhengyang Yang et al. demonstrated notable differences in the baseline gut microbiome between responders and non-responders to PD-1 inhibitor therapy, and developed the SPEED model to predict pCR based on baseline microbial profiles (Yang et al., 2024b). In our study, we also analyzed baseline stool samples from a subset of patients in the PD-1 inhibitor group. However, no significant differences in gut microbiome diversity or abundance were observed between patients who achieved pCR and those who did not, which may be attributed to the limited sample size. Despite this, the potential value of the gut microbiome in predicting immunotherapy response cannot be ruled out. Further studies with larger cohorts are warranted to explore its predictive role. Moreover, dynamic changes in the gut microbiome during treatment should also be emphasized in future research, as they may offer additional insights into the prediction of pCR in the context of neoadjuvant immunotherapy.

There are several limitations in this retrospective study. Firstly, the retrospective nature inevitably limited the statistical power, as the sample size was determined by available clinical data rather than *a priori* calculation. Nevertheless, the observed effect trends were consistent and clinically meaningful, providing a rationale for further validation in larger, well-powered randomized controlled trials. Secondly, long-term follow-up and survival data were not available for comparison. Although the NAR score was used as a surrogate endpoint, it cannot fully substitute for survival outcomes. Thirdly, detailed safety data were not included because of incomplete documentation in the electronic medical record. However, the treatment strategies evaluated in this study have been reported as relatively safe in previous studies.

In recent years, various neoadjuvant strategies have demonstrated promising tumor responses in patients with LARC, but the optimal regimen remains controversial. Future studies should therefore focus on head-to-head comparisons of different neoadjuvant modalities to define the most effective approach. Moreover, in the era of precision medicine, a one-size-fits-all strategy may no longer be appropriate. Stratifying patients according to molecular biomarkers may help optimize treatment selection. In addition, as newer regimens achieve higher complete response rates, nonoperative management strategies like “watch-and-wait” may become increasingly feasible. Thus, improving preoperative assessment to accurately determine tumor response grade will be an important direction for future research.

## Conclusion

In conclusion, we conducted this three-arm retrospective trial comparing the therapeutic efficacy of three common neoadjuvant therapy regimens and found that both TNT and a combination of PD-1 inhibitors yield better tumor responses than traditional nCRT. The diagnostic accuracy of rectal MRI in these new regimens still requires improvement for more effective identification of cCR or ncCR. the gut microbiome shows promise in predicting the effectiveness of immunotherapy for rectal cancer, and we plan to continue conducting relevant studies. We believe that as neoadjuvant therapies evolve, patients will experience improved tumor regression and long-term survival. It is essential that advancements in diagnostic and therapeutic measures, particularly in precision medicine, keep pace with these developments.

## Data Availability

The raw data supporting the conclusions of this article will be made available by the authors, without undue reservation.
