# Supplementary material for: Comparison of currently common neoadjuvant therapy strategies for rectal cancer: a three-arm retrospective study
Source: Front Cell Infect Microbiol. 2025 Dec 9;15:1545195. doi: 10.3389/fcimb.2025.1545195 (PMC12722826; doi:10.3389/fcimb.2025.1545195)

**Supplementary materials**

**Comparison of Currently Common Neoadjuvant Therapy Strategies for Rectal Cancer: A three-arm retrospective study**

Xiao Zhang^1^**^,^** ^δ^, Yang An^1^**^,^** ^δ^, Yuxin Liu^1^**^,^** ^δ^, Ganbin Li^1^, Xiaoyuan Qiu^1^, Yihan Lu, Guole Lin^1,*^

^δ^: Xiao Zhang, Yang An, and Yuxin Liu contributed equally to this work.

**^1^** Department of General Surgery, Peking Union Medical College Hospital, Chinese Academy of Medical Sciences & Peking Union Medical College, Beijing, China

***Corresponding author.**

Prof. Guole Lin (E-mail: [linguole@126.com](mailto:linguole@126.com))

Xiao Zhang^1^**^,^** ^δ^, Yang An^1^**^,^** ^δ^, Yuxin Liu^1^**^,^** ^δ^, Ganbin Li^1^, Xiaoyuan Qiu^1^, Yihan Lu, Guole Lin^1,*^

^δ^: Xiao Zhang, Yang An, and Yuxin Liu contributed equally to this work.

**^1^** Department of General Surgery, Peking Union Medical College Hospital, Chinese Academy of Medical Sciences & Peking Union Medical College, Beijing, China

***Corresponding author.**

Prof. Guole Lin (E-mail: [linguole@126.com](mailto:linguole@126.com))

Figure S1


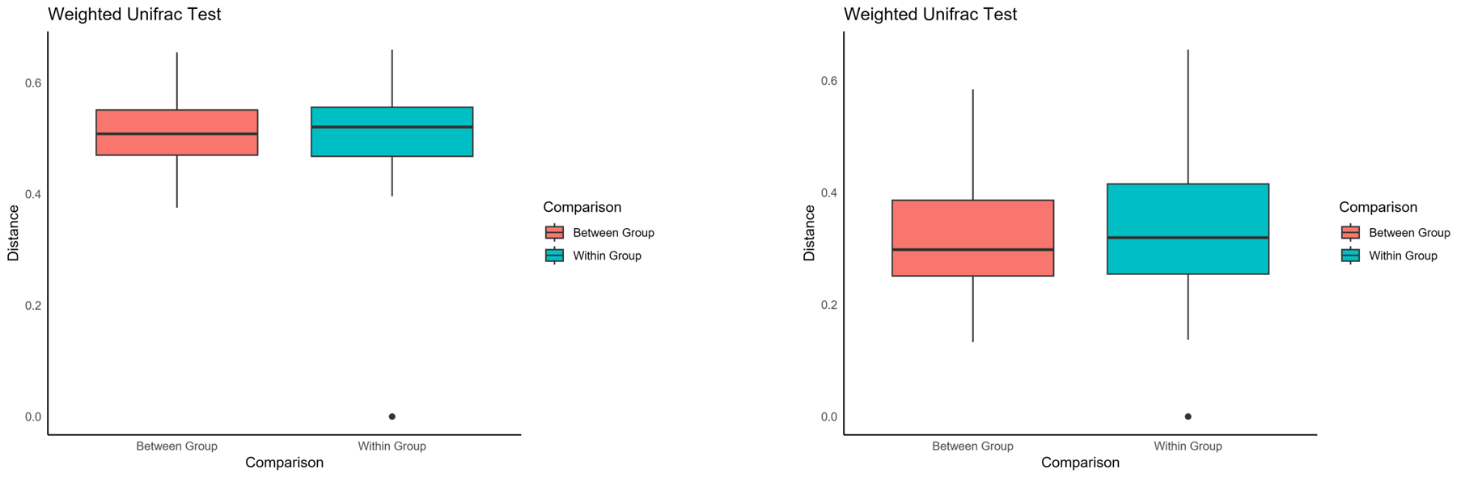

Supplement: Supplementary file 1 [file Table1.docx]
